# Supplementary material for: How leaders are persuaded: An elaboration likelihood model of voice endorsement
Source: PLoS One. 2021 May 18;16(5):e0251850. doi: 10.1371/journal.pone.0251850 (PMC8130952; doi:10.1371/journal.pone.0251850)
Supplement: Attachment — Submitted filename: Response to Reviewers.pdf [file pone.0251850.s003.pdf]

It is a great honor for us that the reviewers provided these valuable comments. The comments indeed inspire us to essentially improve the quality of our research. We carefully studied them and tried our best to make a substantive revision or provided a detailed explanation. The specific revisions we made in response to reviewers' comments are listed as following.

Note: all page numbers mentioned below in relation to our new version of manuscript refer to the manuscript's page numbers with track changes.

Reviewer #1

Comment: *The topic of voice endorsement is very relevant and apt. While the research on voice has been increasing rapidly, the focus on voice endorsement has been relatively sparse. This paper focuses on a very relevant theme and provides insights into the dynamics of voice in organizations. This was an interesting paper and I thoroughly enjoyed reading the paper. The paper is well written and clearly focuses on exploring the context of voice endorsement. The authors leverage the Elaboration Likelihood Model (ELM) to integrate and validate the various aspects related to voice endorsement. The paper has many strengths and is well written. However, I would like to share a few suggestions and concerns that may help improve the paper.*

Responses

We are honored that the reviewer enjoys reading this paper. We are also very grateful that the reviewer read our paper carefully and provided us with detailed revision comments. Next, we will proceed to answer or revise the five comments one by one.

Comment 1: *Page 3, Lines 35-37*

"Among research studies, researchers have mainly focused on how message factors influence voice endorsement and have ignored sender and receiver factors.

This statement may be rephrased to say "mostly ignored" or "not focussed on", instead of "have ignored". For example, Whiting et al. (2012) and Lam et al. (2019) and have considered sender aspect (i.e., voicer credibility), and Schreurs et al. (2020) have considered relational aspects of sender and receiver (i.e., psychological distance). The authors may also benefit by referring to Ng et al. (2019).

Ng, K. Y., Van Dyne, L., & Ang, S. (2019). Speaking out and speaking up in multicultural settings: A two-study examination of cultural intelligence and voice behavior. Organizational Behavior and Human Decision Processes, 151, 150-159.

Responses

We agree with the reviewer that the recommended statement *"most ignored"* is more accurate in expressing the lack of current research. We have rewritten this sentence, and

we thank the reviewer for this suggestion on the wording. Besides, we appreciate the reviewer for providing the reference of Ng et al. (2019), which is an influencing study in voice research. We cited this reference in the place where we tried to emphasize the importance of employee voice for the organization (see Page 2). The specific revisions are listed below or can be found on Page 3

Specific revisions

Although voice endorsement is important, empirical research is limited. Among research studies, researchers mainly focused on how message factors influence voice endorsement and mostly ignored sender and receiver factors.

Comment 2: *Pages 5-6, Lines 103-107*

"In prior research, most scholars believed that the influence of mood could be transferred only from high-power to low-power individuals rather than the other way around. That belief led to extensive efforts to determine the role of employee mood in upward communication [e.g., 18,19] and little attention to leader mood in downward communication."

> *The research cited are examples of "leader mood in downward communication". These citations suggest that you were trying to argue the opposite. Please check and rephrase the above sentence appropriately to clearly convey your argument.*

Responses

We got it wrong. We are very grateful that the reviewer read our article so seriously and carefully, otherwise we would have sent the wrong message to our readers. The specific revisions are listed below or can be found on Page 5.

Specific revisions

In prior research, most scholars believed that the influence of mood could be transferred only from high-power to low-power individuals rather than the other way around. That belief led to extensive efforts to determine the role of leader mood in upward communication of employee voice [e.g., 18,19] and little attention to employee mood in downward communication of voice endorsement.

18. Liu W, Tangirala S, Lam W, Chen Z, Jia RT, Huang X. How and when peers' positive mood influences employees' voice. Journal of Applied Psychology. 2015;100: 976–989. doi:10.1037/a0038066

19. Liu W, Song Z, Li X, Liao Z. Why and when leaders' affective states influence employee upward voice. Academy of Management Journal. 2017;60: 238–263. doi:10.5465/amj.2013.1082

Comment 3: *Page 21, Line 346*

"Prior work on voice has begun to explore employee mood but has tended to minimize leader mood."

Please elaborate what you mean by "tended to minimize leader mood".

Responses:

Thanks to the reviewer for noticing this point. It is true that this statement does not express itself clearly. We were trying to convey that "tended to minimize the role of leader mood in voice research". Specifically, most of the research focused on the effect of leader mood on voice communication, as scholars believed that mood transmission was a one-way process; that is, mood from high-power individuals affected low-power individuals, and vice versa did not hold true. In our study, we challenged this premise by arguing that the transmission of mood is a two-way process, i.e., the positive mood of low-power employees can also influence the decision-making behaviors (voice endorsement) of their high-power leaders."

Many thanks to the reviewer for pointing out this poorly expressed argument. We have elaborated on it in the new version of our manuscript. The specific revisions are listed below or can be found on Page 24.

Specific revisions

Prior work has begun to explore the importance of employee mood in the communication process of voice. However, most of these studies assumed that the transmission of mood was a one-way process in which high-power individuals influence low-power individuals and thus did not realize the importance of employee mood in leaders' decision-making behaviors.

Comment 4: *Page 23, Lines 383-393*

The authors may benefit by referring to Ward et al. (2016) in relation to their discussion on impact of high-context and low-context cultures on voice.

Responses

Many thanks to the reviewer for recommending this article. The article, published in the top-tier journal, provides a sound theoretical basis for our paper in understanding the impact of high-/low-context cultures on communication processes. By citing the literature, we further clarify how high-context culture might influence our findings. As the reviewer stated, we do gain benefit by referring to Ward et al.'s (2016) paper. The specific revisions are listed below or can be found on Page 27.

Specific revisions

In high-context societies such as China, individuals are more likely to focus on (and make use of) mood because their main goal in interactions is to preserve harmony and

save face for others [47]. Meanwhile, individuals in high-context cultures rely more on shared understandings (contexts) to convey information, so they tend to be more comfortable with ambiguous messages and less emphasis on the content of the information being exchanged [47]. Thus, compared to respondents from low-context cultures, the leader respondents in the present study may be more sensitive to employee mood and less sensitive to employee credibility, resulting in the impact of positive mood being magnified and the impact of voicer credibility being reduced. Therefore, future research can be conducted in low-context cultures to examine whether our findings remain valid or introduce the low-/high-context cultures as a moderating variable to explore its impact on voice endorsement.

Comment 5: *I hope that these suggestions and concerns may help the authors revise and improve the paper.*

Responses

We thank the reviewer very much. These suggestions above have helped us to improve a lot in terms of wording, error correction, and theoretical interpretation. Thank you :)

Reviewer #2

Comment: *The research was conducted well, and it is a unique approach to understand about how voice endorsement can influence other people.*

Responses: We thank the reviewer for the recognition of our paper.

Comment 1: *You will need to defend why 168 paired samples is enough? Does it represents the overall populations?*

Responses

Thank you for raising this question. To figure out the appropriate sample size needed for linear regression, we read a few articles and found that scholars have different views on the rule of thumb for sample sizes. For example, Gefen, Straub, and Boudreau (2000, p9) suggested that linear regression supports smaller sample sizes and that the minimal sample size required is at least 30. According to Burmeister and Aitken (2012, p8), the sample size for regression analysis can use the 20:1 rule, which states that the sample size ratio to the number of parameters in a regression model should be at least 20 to 1. If we adopt this rule, our study's sample size would be 60 [(4-1) x 20 = 60]. Similarly, Green (1991, p504) proposed an alternative sample size calculation method for multiple regression as N > 50 + 8p, where p is the number of predictors. Our study has four predictors, indicating a sample of 88 needed for the linear regression.

Besides, we reviewed some articles in top journals and found that the sample size requirement for regression analysis is not very demanding. For example, in Ward et al.'s (2016, p8) study, they collected 131 paired samples (131 subordinates and 131 managers) to run OLS regression analyses. Similarly, in Li et al.'s (2019, p877) study, they conducted a series of linear regressions using a sample of 198 managers. As another example, the sample size Liu et. al (2015, p981) used in their OLS regression analysis was 142. Based on the collected information, we believe that a sample size of 168 could support our regression analysis for the present study.

The sample's representativeness is usually influenced by three factors: randomness, sample size, and sampling approach. When designing the study, we considered collecting questionnaires from companies with different employee sizes, different products, and different stages of development. We also tried to seek the variability of questionnaire respondents (e.g., age, gender, and years of work experience) to increase the sample's representativeness. For the sample size, the larger the sample size is, the more representative of the overall population. Although we discussed above that a sample of 168 is sufficient for regression analysis, we agree with the reviewer that larger sample size could increase our sample's representativeness.

We acknowledge some imperfections in the representativeness of our sample, but we

find that the results of our hypothesis remain significant even after controlling for the seven control variables. Therefore, we have reason to believe that such imperfections in our sample's representativeness do not have a significant impact on our estimation results. The specific revisions are listed below or can be found on Page 9.

Specific revisions

Based on previous studies [31,32], a sample of 168 is sufficient to perform the regression analysis.

1. Gefen D, Straub D, Boudreau M-C. Structural Equation Modeling and Regression: Guidelines for Research Practice. CAIS. 2000;4. doi:10.17705/1CAIS.00407 1.
2. Burmeister E, Aitken LM. Sample size: How many is enough? Australian Critical Care. 2012;25: 271–274. doi:10.1016/j.aucc.2012.07.002
3. Reynolds N, Diamantopoulos A, Schlegelmilch B. Pre-Testing in Questionnaire Design: A Review of the Literature and Suggestions for Further Research. Market Research Society Journal. 1993;35: 1–11. doi:10.1177/147078539303500202
4. Ward A-K, Ravlin EC, Klaas BS, Ployhart RE, Buchan NR. When do high-context communicators speak up? Exploring contextual communication orientation and employee voice. Journal of Applied Psychology. 2016;101: 1498–1511. doi:10.1037/apl0000144
5. Li J (Jason), Barnes CM, Yam KC, Guarana CL, Wang L. Do not like it when you need it the most: Examining the effect of manager ego depletion on managerial voice endorsement. J Organ Behav. 2019;40: 869–882. doi:10.1002/job.2370
6. Liu W, Tangirala S, Lam W, Chen Z, Jia RT, Huang X. How and when peers' positive mood influences employees' voice. Journal of Applied Psychology. 2015;100: 976–989. doi:10.1037/a0038066

Comment 2: *You will also need to inform about the data collection process in the study? How long? How did you get those data?*

Responses: This is good advice. We have built on our previous work to provide a more detailed description of the data collection process, particularly in terms of the time schedule and how we collected the questionnaires. The specific revisions are listed below or can be found on Page 8-9.

Specific revisions

From April to June 2019, we contacted 200 team leaders (or department managers) working for 73 firms in Zhejiang, China. Survey data were collected mostly from cities

of Wenzhou, Yiwu, and Taizhou, where the manufacturing industry was more developed (the data used in the present study were part of a broader data collection effort). We used a paired-questionnaire survey design. During the specific process of data collection, we usually invited all participants (4 to 6 participants per company in general) from the company to a nearby conference room, explained how to fill out the questionnaire with explicit instructions, and promised the confidentiality of all individual responses to reduce their worries about information leakage. For each matched pair of leader and employee, we would distribute two different envelopes, one containing the leader version of the questionnaire and the other containing the employee version of the questionnaire.

We then gave all participants adequate time to complete their questionnaires, which they put into the sealed envelopes thereafter. Each employee completed items related to his/her own voicer credibility, positive mood, and demographic information. Each employee's supervisor provided evaluations of his or her voice endorsement, felt obligation, cognitive flexibility, and demographic information. In total, we sent out 400 questionnaires (200 for leaders and 200 for employees) and received responses from 173 leaders (86.5% response rate) and 182 employees (91.0% response rate)). After the deletion of invalid or unmatched questionnaires, we finally obtained 168 dyads. Based on previous studies [30,31], a sample of 168 is sufficient to perform the regression analysis of the present study.

Comment 3: *You will need to defend on why you use odd number of Likert Scale. Some researchers like presented in the study of Dhar and Simonson (2003), encourage even number of Likert scale to avoid middle answers.*

Responses: That is an excellent question that we had not noticed before. In our previous study, we simply borrowed or adapted scales developed by other domain researchers without considering the possible impact of the option settings on statistical results. We really learned a lot by reading the study of Dhar and Simonson (2003) referred by the reviewer.

After careful reading and discussion, we believe that the main findings of Dhar and Simonson's (2003) work support us to use an odd number of Likert Scale. According to them, the no-choice option is usually a better choice because it helps resolve the preference uncertainty and discomfort associated with forced choice in competition with other conflict resolutions (P148), while forced-choice procedures may often produce biased or incomplete findings that lead to incorrect conclusions (P156). Other related statements from this recommended study are list below.

> P158: *The results also apply to other choice domains. For example, polls of voting intentions are sometimes conducted sing a design in which voters are given the "no opinion/don't know" option, whereas in other polls they are forced to express a preference among the available candidates (Krosnick*

> *2000). The current results suggest that the latter forecasts may often be systematically biased.*
>
> *Our results also have implications for choice-based conjoint analysis. For example, the Sawtooth software allows a researcher to include a "choose none" option when conducting a conjoint study. Our results suggest that including such an option can make the choice task more realistic and the experience more pleasant for the respondent.*

In our study, the odd-numbered Likert scale we used was a type of no-choice option settings in which we provide the choice of "neither agree nor disagree/uncertainly." Our participants did not necessarily have to choose between disagree and agree (1 = strongly disagree, 2 = disagree, 3 = neither agree nor disagree/uncertainly, 4 = agree, 5 = strongly agree). In addition, using an odd-numbered Likert scale is a relatively common practice in psychological research. For example, Lam and Lee (2019, P652) measured their research constructs (e.g., voicer credibility) on a five-point scale ranging from 1 (strongly disagree) to 5 (strongly agree). Liang et.al (2012) used scales ranging from 1 (strongly disagree) to 5 (strongly agree) for all their variables (e.g., employee voice).

Overall, we thank the reviewer for offering such a good reference. By studying it, we have thought more about the theoretical aspects of the scale items' setting.

1. Dhar R, Simonson I. The Effect of Forced Choice on Choice. Journal of Marketing Research. 2003;40: 146–160. doi:10.1509/jmkr.40.2.146.19229
2. Lam CF, Lee C, Sui Y. Say it as it is: Consequences of voice directness, voice politeness, and voicer credibility on voice endorsement. Journal of Applied Psychology. 2019;104: 642–658. doi:10.1037/apl0000358
3. Liang J, Farh CIC, Farh J-L. Psychological Antecedents of Promotive and Prohibitive Voice: A Two-Wave Examination. AMJ. 2012;55: 71–92. doi:10.5465/amj.2010.0176

Comment 4: *Is there any practical contributions that can be presented from this study? What are the benefits for the businesses and practitioners of your work?*

Responses: We totally agree with this comment that we should provide practical contributions to our study. We have added the practical implications of our study from employee and manager perspectives in the part of *Theoretical and Practical Contributions*. The specific revisions are listed below or can be found on Page 26-27.

Specific revisions

Our findings also offer valuable practical implications for both employees and managers. From the employee perspective, it will be essential to understand how they

present themselves will affect their leaders' cognitive or affective processes of voice endorsement. Building credibility often takes a lengthy period and many work-based interactions, and it is difficult for employees to change their leaders' perception of trust in them within a short time. Therefore, one possible action strategy they can employ is to present positive emotions towards their work, colleagues, and supervisors if they expect their voice to be taken. Meantime, our results also suggested that leaders' motivation and ability are important moderators of the associations between voicer credibility (positive mood) and managerial endorsement, so employees should get to know their leaders before speaking up. For example, if an employee notice that his or her leader is a person who can handle complicated issues but lacks a sense of obligation to the organization, they must be aware that showing positive mood may have little impact on their leader's voice endorsement. The employee should be cognizant of their credibility before speaking up. Especially for those less credible employees, it may be better to build their credibility first. From the manager or organizational perspective, they usually expect their voice endorsement to be objective and fact-based rather than driven by emotions. In this case, the organization should increase managers' sense of responsibility for the organization or their cognitive ability to address complex issues through management efforts, such as carrying out regular communication meetings on organizational goals or theoretical training activities that combine company practices.

Comment 5: *Related with the practical contributions, it can also be explored on the combination of not just voice alone, but perhaps with other traits such as body gestures, linguistic traits, selections the combinations of the words, and other factors.*

Responses

Many thanks for this valuable comment. The reviewer offered a good direction on what factors should be explored in the future research of voice endorsement. We agree with the reviewer that future research could also examine the impact of employees' body gestures, linguistic traits, or selection (combination) of the words on their leaders' voice endorsement. The specific revisions are listed below or can be found in the part of *limitations and future research* (Page 28).

Specific revisions

In addition, we encourage research to explore other determinants of voice endorsement based on the perspective of ELM. For example, we examined only positive mood as a peripheral cue in our study. However, as another peripheral cue, negative employee mood could also influence leader voice endorsement because it can trigger affective responses such as defense and fear [19]. Besides employee mood, other peripheral cues such as employees' body gestures, linguistic traits, and the combinations (selections) of the words may also influence the voice endorsement of their leaders and is worthy of further investigation.

Reviewer #3

Comment: *Quiet an interesting study. The authors have explored a very unique area and the arguments and conclusions are well noted. The limitations and theoretical contributions are well noted. The study has more room for further research on this area and scope.*

Responses:

Many thanks for the recognition of our paper.

Reviewer #4

Comment: *I want to thank the authors for their effort in providing this manuscript titled "How leaders are persuaded: an elaboration likelihood model of voice endorsement." I reviewed the manuscript with great interest as I feel this is an exciting area of research. In their manuscript, the authors provided an excellent background and a comprehensive review of the literature. In particular, I think the way they approached each hypothesis was easy to follow and to get a good idea about gaps in literature they want to cover. However, I find myself disappointed with the statistical analysis they provided, considering the effort they show in this manuscript.*

Responses: We feel honored that the reviewer was interested in and recognized the theoretical values of our paper. However, we are also truly sorry that our data analysis did not meet the reviewer's expectation. To address the reviewer's concerns, all our authors carefully studied and discussed each of the reviewer's comments and provided detailed explanation and substantive revisions to all questions. We hope that the efforts we have made on the new version of the manuscript can convince the reviewer.

Comment 1: *In the section "common method bias," the authors described their ex-ante steps taken to minimize bias per Podesakoff et al. (2003).[1] Additionally, they mentioned using "commonly used" Harman's single factor test as an ex-post remedy. However, several publications and simulations have provided evidence against using such a test.[2,3] Therefore, the authors' confirmation about the absence of CMB in their research can be questionable if they are using this test alone to provide such a sense of security.*

Responses

As the reviewer noted, we were aware of CMB's possible negative impact on our study in advance and took an ex-ante steps (i.e., multiple sources), which we think was the most effective way to reduce the CMB problem. Any other statistical techniques on CMB testing can only check (not reduce) whether the CMB will have a significant impact on the results.

However, we fully agree with the reviewer that the CMB testing with Harman's single factor is simple to perform but was criticized by many researchers. Therefore, we added two other techniques (CFA and ULMC) to further demonstrate that our study's common method bias was not severe. In particular, the ULMC method is superior to the CFA method, and the CFA method is superior to Harman's single factor. We are very grateful to the reviewer for raising this issue, as we have learned two new CMB-check techniques. The specific revisions are listed below or can be found on Page 13-14.

Specific revisions

Third, we further carried out three different tests to verify that the CMB does not significantly influence the stability of our parameter estimates. (1) Harman's single factor test. The result shows that the variance for the first factor is 34.00% (< 40%) [40], suggesting that CMB is not a major issue in the present study. (2) CFA approach. The CFA method in testing CMB considers that if the fit indicators of the single-factor CFA model do not meet the criteria of good fit, or if the single-factor CFA model is the worst-fitting model among the competing models, it means that the CMB is not serious [41]. As shown in Table 2, all indicators of the single-factor model (χ2 /df = 6.81, RMSEA = 0.187, CFI = 0.55, IFI = 0.55, TLI = .50, and SRMR = 0.159) failed to meet the requirements and fit worse than the other five competing models. (3) ULMC Technique. To use the ULMC Technique, Richardson, Simmering, and Sturman (2009) suggested that researchers could first construct a method factor relating to all items in the hypothesized model. Then, researchers compare this new model with the hypothesized model [42]. If the variance of the two models is significantly different, then the CMB is severe, otherwise, the CMB is not severe. Following the procedures, we found that the variance between the two models is not significant [1.15, $\Delta\chi^2/\Delta df$ = (372.97-346.49)] / (199-176]], suggesting that the CMB is not severe in our study.

Comment 2: *Table 3, from a statistical point of view, I am not convinced that producing mean, sd, and correlations for categorical data is sensible. I understand that many papers have been published with such tables, but I do not find such tables provide either correct information or have added value. I believe this table should not be included in the analysis in its current form. Frequencies, percentages, tests of associations, even if we want to use correlations as mentioned in the table, that type of correlation should be indicated.[4]*

Responses

We are very grateful to this reviewer for this to-the-point comment. As the reviewer stated, we simply followed the "rules of the field" when providing this table without thinking about the value we would add. In our research field of psychology, most researchers would provide such a table “descriptive statistical analysis” without asking why it was done.

We agree with the reviewer that a table containing frequencies and percentages would be more valuable than that containing means and sd because the former can provide more detailed information to reveal the sample attributes such as distribution type, concentration/dispersion trends, and certain outliers. The specific revisions are listed below or can be found on Page 9-10.

Specific revisions

Table 1 shows some basic information about the sample. As indicated below, most participants who filled out the questionnaires were male. Specifically, the proportion of male employee participants is 64.3%, and that of male leader participants is 74.4%. From the categories of organization tenure, we can find that the majority of employee

participants have been working at their companies for less than three years (a proportion of 54.2%), while that of leader participants is for more than three years (a proportion of 72.1%). Regarding the education level, a large proportion of both employee (50.6%) and leader participants (34.5%) are in college, although the latter has a relatively higher percentage of higher education (more than college) than the former.

Table 1. Background information of the employee and leader participants

Demographic variables	Category	Frequency	Percentage
Employee's gender	Male	108	64.3%
	Female	60	35.7%
Employee's organization tenure	Within 1 year	50	29.8%
	1~3 years	41	24.4%
	3~8 years	32	19.0%
	More than 8 years	45	26.8%
Employee's education	middle school or below	33	19.6%
	high school	15	8.9%
	junior college	21	12.5%
	college	85	50.6%
	more than college	14	8.3%
Leader's gender	Male	125	74.4%
	Female	43	25.6%
Leader's organization tenure	Within 1 year	13	7.7%
	1~3 years	34	20.2%
	3~8 years	69	41.1%
	More than 8 years	52	31.0%
Leader's education	middle school or below	5	3.0%
	high school	21	12.5%
	junior college	58	34.5%
	college	58	34.5%
	more than college	26	15.5%

Comment 3-1: *The authors used CFA and path modeling to explain the causal relationship in their analysis and provide evidence for their hypothesis testing. However, they are not providing a graphical representation of these relationships. They shifted to OLS and hierarchical regression analyses. Such a method is a big assumption from their side that the response variable is continuous.*

Responses

First, we would like to clarify that we only used CFA analysis but not path modeling. The two types of analyses can be used separately. For example, Lam, Lee, and Sui (2019) only used CFA to ensure that the multiitem measures they used could be appropriately modeled as distinct constructs. Our purpose using CFA is the same as theirs. Second, we thank the reviewer for this comment which offers an opportunity to explain why we used linear regression rather than the SEM techniques.

In recent years, the growing interest in the SEM technique and recognition of its importance in research suggests the need to compare it with other statistical techniques so that research designs can be selected appropriately. Compared with linear regression, the SEM technique does have several unique advantages in handling some complicated research models, such as models that contain multiple dependent variables. The SEM technique can also provide a holistic analysis, including structural and measurement models, to estimate a series of interrelated dependence relationships simultaneously. However, there are distinct differences between SEM and regression that make each more or less appropriate for certain analysis types. Thus, choosing an analysis method correctly based on the research objectives is crucial. Next, we will explain why regression would be a more appropriate choice than SEM in our study regarding data distribution and the sample size.

(1) Distribution issues. One primary concern when using SEM path modeling is that the hypothetical variables should be normally distributed (multivariate normal distribution). In Holland et al.'s (2017, p717) study, they conducted a literature analysis on 78 papers published in four top journals that includes both moderation and mediation and found that only 5% of these papers were statistically analyzed using any type of SEM. One possible reason is that the product-indicator approaches used by SEM to analyze moderation are flawed. Specifically, the product-indicator approaches use the product of the assessed independent and moderating variables as an indicator of the interaction term. However, this interaction term is non-normal distributed, which violates the data assumptions of SEM. In our study, we have four moderating effects. If these four moderations are included in a path model (SEM) simultaneously, it can cause significant bias in the estimation of the study results.

Furthermore, the regression analysis procedure of moderating effects is relatively simpler than that of SEM. One adds a new variable to the regression model, calculated as the product of the assessed independent variables that are assumed to interact, and then rerun the regression. However, this procedure does not work well in SEM because

such a calculated interaction term will have a high shared residual variance with the variables from which it is derived (Gefen et al., 2000).

(2) Sample size issues. Another concern when using SEM is the requirement of a high sample size. In a top management journal, Shah and Goldstein (2006) suggested that the sample size issue is one of the main concerns in selecting SEM and regression analysis, as it would impact the reliability of parameter estimates and statistical power. Moreover, they further indicated that to reach robust results in SEM, the minimal sample size is ten times the number of items in the research model, which is at least a sample of 310 (10 × 31 items) in our study. Obviously, our current sample size is not sufficient nor suitable to run the path analysis of SEM.

Overall, the use of regression in this study can better match the characteristics of the data distribution and the sample size, and the procedures for testing moderation are simpler and more effective. In recent years, research using regression analysis as an empirical approach has been published in many leading academic journals (e.g., Allison et al., 2017, Lam et al., 2019), which also provides some evidence for the validity and legitimacy of the traditional statistical method, i.e., regression. We hope that our explanation of why we use regression (instead of path modeling in SEM) as our preferred method can address the reviewer's concerns.

1. Holland, S. J., Shore, D. B., & Cortina, J. M. (2017). Review and recommendations for integrating mediation and moderation. *Organizational Research Methods*, *20*(4), 686–720.
2. Gefen, D., Straub, D., & Boudreau, M.-C. (2000). Structural Equation Modeling and Regression: Guidelines for Research Practice. *Communications of the Association for Information Systems*, *4*.
3. Shah, R., & Goldstein, S. M. (2006). Use of structural equation modeling in operations management research: Looking back and forward. *Journal of Operations Management*, *24*(2), 148–169.
4. Allison TH, Davis BC, Webb JW, Short JC. Persuasion in crowdfunding: An elaboration likelihood model of crowdfunding performance. Journal of Business Venturing. 2017;32: 707–725.
5. Lam CF, Lee C, Sui Y. Say it as it is: Consequences of voice directness, voice politeness, and voicer credibility on voice endorsement. Journal of Applied Psychology. 2019;104: 642–658.

Comment 3-2: *There is no measuring for the suitability of using OLS in the framework of their analysis. The authors did not check for OLS assumptions.*

Responses

Many thanks to the reviewer for raising this issue. It is true that, like many previous studies, we did not conduct ex-ante tests of the suitability of whether linear regression could be used. In the new version of the manuscript, we tested the assumptions of linear regression, and the results showed that we could use this method. Specifically, we examined four aspects: multicollinearity, homoskedasticity, normality of the residual, and autocorrelation before running OLS regression.

(1) Multicollinearity. When multicollinearity is observed, the association between the variables leads to larger standard deviations and wide confidence intervals for the results. We can identify whether multicollinearity is severe by looking at the variance inflation factor (VIF) between variables. In our study, the maximum value of VIF is 1.55 (<10), suggesting that the multicollinearity problem does not significantly influence the stability of the parameter estimates (Dielman, 1991).

Dielman TE. Applied Regression Analysis for Business and Economics. Boston, MA: PWS-Kent; 1991.

(2) Homoskedasticity. Homoscedasticity is one of the essential assumptions of linear regression, which refers to the fact that the random error terms in the overall regression function have the same variance. Generally, we can graphically observe whether there is an evident pattern in the distribution of the variance. If there is no obvious pattern in the distribution, the homoscedasticity assumption of linear regression is satisfied, and the regression analysis can be performed. As shown in the figure below, we can see no significant pattern in the distribution of the variance, so it is safe to run a linear regression.

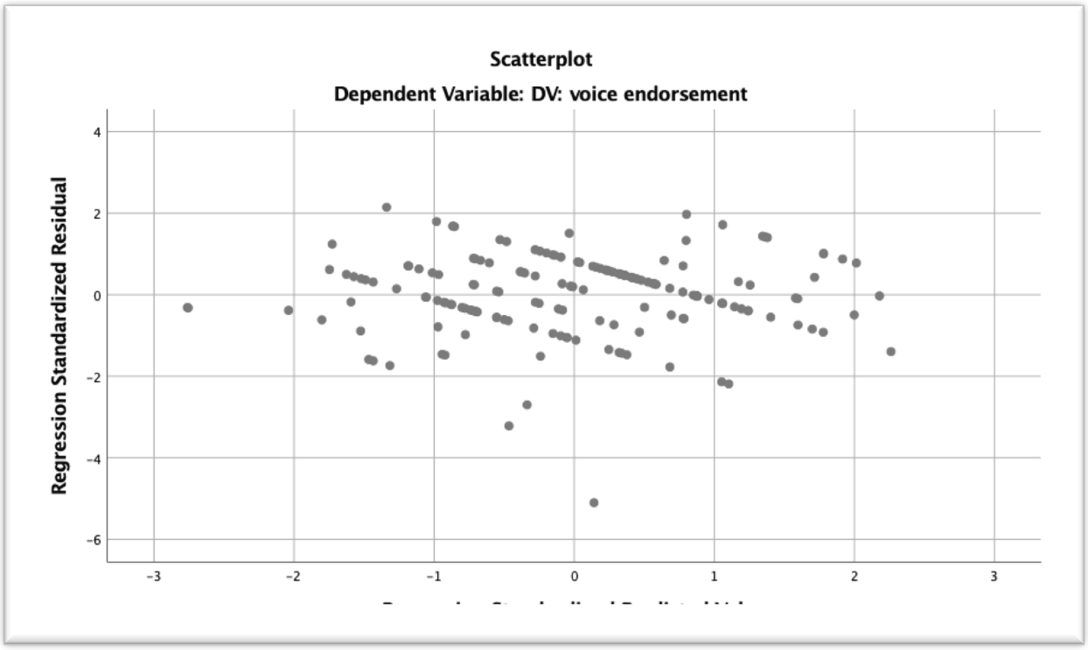

(3) Normality of the residual. The third important assumption of linear regression is that the error term should obey a normal distribution. Otherwise, the confidence intervals for the estimated statistical results can become highly unstable. In our study, we used a normal P-P Plot and histogram to observe the normality of the residual. As shown in the two figures below, the P-P Plot plots fall approximately on a straight line, and the frequency of the regression standardized residual presents an excellent normal distribution. Therefore, it is safe to run OLS linear regression.

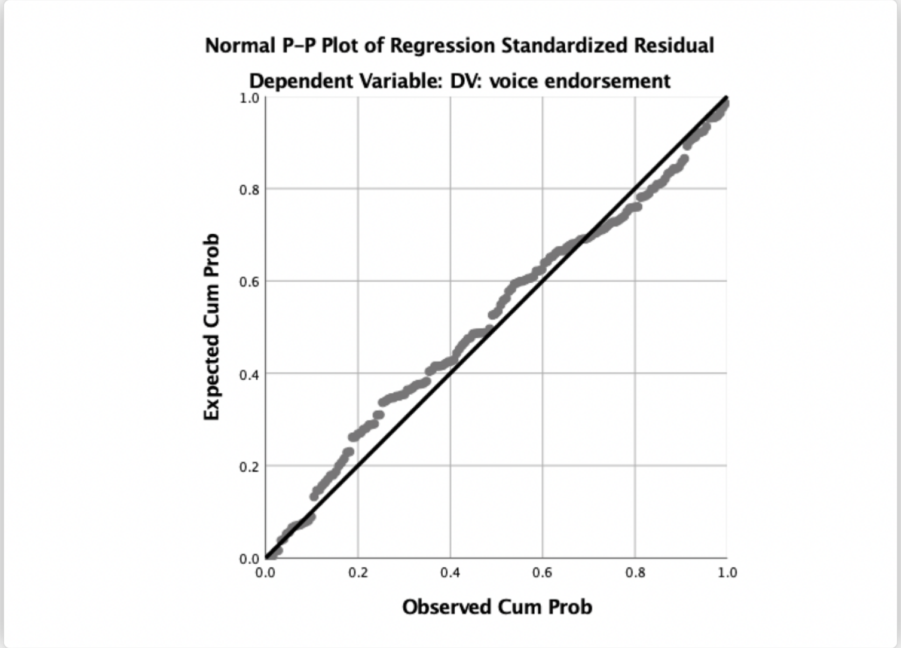

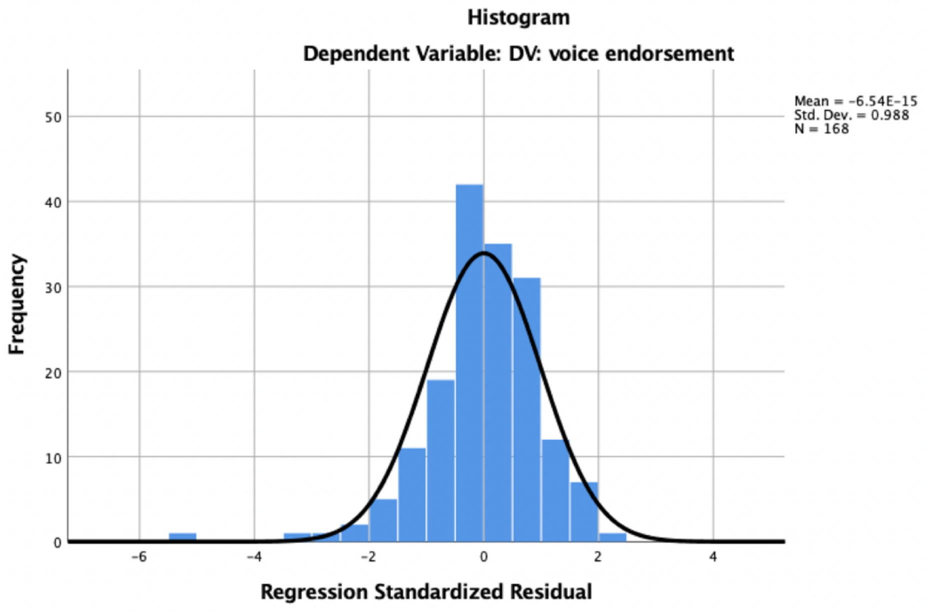

(4) Autocorrelation. When autocorrelation occurs, the standard deviation measured tends to be smaller, leading to narrower confidence intervals. According to Durbin-Watson (DW) checks, the closer the DW is to 2, the greater the certainty of determining non-autocorrelation. And if d_u < DW= < 4 - d_u, then we can conclude no autocorrelation there. By running DW tests in regression analysis, we found that our DW value is 2.14 and the maximum d_u is 1.11. Therefore, our DW value is very close to 2, and it is between d_u and 4 - d_u. We conclude that there is no autocorrelation in our regression analysis.

Model Summary[b]

Model	R	R Square	Adjusted R Square	Std. Error of the Estimate	Durbin-Watson
1	.706[a]	.498	.486	.51812	2.135

Overall, we checked our data's OLS assumptions by testing the multicollinearity, homoskedasticity, normality of the residual, and autocorrelation. Once again, we thank the reviewer for providing us with this learning opportunity, which allowed us to develop a deeper understanding of linear regression. The specific revisions are listed below or can be found on Page 19.

Specific revisions

We employed ordinary least squares (OLS) regression analysis to evaluate the hypotheses. Before running the regression, we checked the linear regression assumptions in terms of multicollinearity, homoskedasticity, normality of the residual, and autocorrelation. The results showed that our data characteristics meet all the assumptions of the linear regression. (1) Multicollinearity. When multicollinearity is observed, the association between the variables leads to larger standard deviations and wide confidence intervals for the results. In our study, the maximum value of variance inflation factor (VIF) is 1.55 (<10), suggesting that the multicollinearity problem does not significantly influence the stability of the parameter estimates. (2) Homoskedasticity. Homoscedasticity is one of the essential assumptions of linear regression, which refers to the fact that the random error terms in the overall regression function have the same variance. In our study, we found that there is no obvious pattern in the distribution of the variance. (3) Normality of the residual. The third important assumption of linear regression is that the error term should obey a normal distribution. Otherwise, the confidence intervals for the estimated statistical results can become highly unstable. In our study, the P-P Plot plots fall approximately on a straight line, and the frequency of the regression standardized residual in the histogram presents a good normal distribution. (4) Autocorrelation. When autocorrelation occurs, the standard deviation measured tends to be smaller, leading to narrower confidence intervals. In our study, the Durbin-Watson (DW) value is 2.14 (close to the expected value of 2). A further calculation showed that it is between d_u and 4 - d_u, indicating no autocorrelation in our

regression analysis.

Comment 4: *The concepts the authors are measuring are derived from latent variables that cannot be measured using the methods suggested in their analysis approach. Methods such as SEM and IRT should have been explored in the context of questionnaire data analysis. Moreover, I am not sure what is the response variable in their analysis using OLS. How the authors pooled the results into a single response variable?!*

Responses

Many thanks for raising these valuable comments. We have elaborated on the reviewer's concern about the SEM issue on Comment 3-1. Next, we mainly focus on the second point of the reviewer's concerns relating to the response variable.

In our study, the dependent variable is voice endorsement, the independent variables are voice credibility and positive mood, the moderated variables are felt obligation and cognitive flexibility, which can be more clearly identified in the figure below. To further clarify how we used the hierarchical regression analysis to pool the results together, we provided a summary description of how the eight models in Table 4 were developed. The specific response variables in each model are presented below.

Model 1: Dependent variable (voice endorsement) + All control variables (e.g., employee and leader gender).

Model 2: Dependent variable (voice endorsement) + Independent variable (voicer credibility) + All control variables.

Model 3: Dependent variable (voice endorsement) + Independent variable (positive mood) + All control variables.

Model 4: Dependent variable (voice endorsement) + Two independent variables (voicer credibility & positive mood) + All control variables.

Model 5: Dependent variable (voice endorsement) + Two independent variables (voicer credibility & positive mood) + moderated variable (felt obligation) + moderating effect (voicer credibility × felt obligation) + All control variables.

Model 6: Dependent variable (voice endorsement) + Two independent variables (voicer credibility & positive mood) + moderated variable (felt obligation) + moderating effect (positive mood × felt obligation) + All control variables.

Model 7: Dependent variable (voice endorsement) + Two independent variables (voicer credibility & positive mood) + moderated variable (cognitive flexibility) + moderating effect (voicer credibility × cognitive flexibility) + All control variables.

Model 8: Dependent variable (voice endorsement) + Two independent variables (voicer credibility & positive mood) + moderated variable (cognitive flexibility) + moderating effect (positive mood × cognitive flexibility) + All control variables.

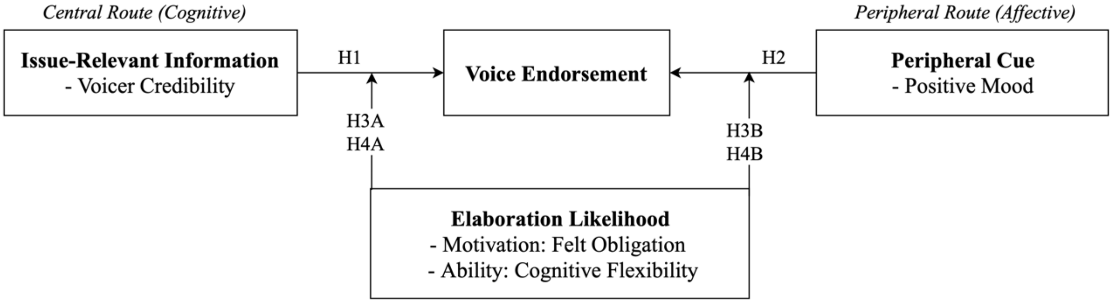

Overall, we performed a linear regression for each of the models, i.e., eight linear regressions in total. We recorded the results we needed for each regression we ran and then pooled them into one table. The specific revisions are listed below or can be found on Page 19-20.

Specific revisions

The results are presented in Table 4. Model 1 includes only the dependent variable and control variables. Models 2 includes the dependent variable, the independent variable of voicer credibility, and the control variables. Model 3 includes the dependent variable, the independent variable of positive mood, and the control variables. Model 4 includes the dependent variable, the two independent variables, and the control variables. Model 5 includes the dependent variable, the two independent variables, the moderated variable of felt obligation and its moderating effect with voicer credibility, and the control variables. Model 6 includes the dependent variable, the two independent variables, the moderated variable of felt obligation and its moderating effect with positive mood, and the control variables. Model 7 includes the dependent variable, the two independent variables, the moderated variable of cognitive flexibility and its moderating effect with voicer credibility, and the control variables. Model 8 includes the dependent variable, the two independent variables, the moderated variable of cognitive flexibility and its moderating effect with positive mood, and the control variables.

Comment 5: *I find the data's analysis inadequate to the research and the type of data the authors provided. Therefore, I feel that this manuscript in it is current form is not suitable for publication. The data should be re-analyzed using more statistically sound approaches.*

Responses

Once again, we would like to thank the reviewer for questioning our method and providing constructive comments. During the revisions, we learned some important points that we had not paid attention to. We deeply felt that our method analysis had become more rigorous by following the reviewer's advice. We hope that our above

revisions could address most of the reviewer's concerns on our data analysis. Thank you.
