# Supplementary material for: How leaders are persuaded: An elaboration likelihood model of voice endorsement
Source: PLoS One. 2021 May 18;16(5):e0251850. doi: 10.1371/journal.pone.0251850 (PMC8130952; doi:10.1371/journal.pone.0251850)
Supplement: Attachment — Submitted filename: Reviewer Comments - PlosOne.pdf [file pone.0251850.s002.pdf]

How leaders are persuaded: an elaboration likelihood model of voice endorsement

REVIEWER COMMENTS:

The topic of voice endorsement is very relevant and apt. While the research on voice has been increasing rapidly, the focus on voice endorsement has been relatively sparse. This paper focuses on a very relevant theme and provides insights into the dynamics of voice in organizations. This was an interesting paper and I thoroughly enjoyed reading the paper. The paper is well written and clearly focuses on exploring the context of voice endorsement. The authors leverage the Elaboration Likelihood Model (ELM) to integrate and validate the various aspects related to voice endorsement. The paper has many strengths and is well written. However, I would like to share a few suggestions and concerns that may help improve the paper.

Page 3, Lines 35-37:
"Among research studies, researchers have mainly focused on how message factors influence voice endorsement and have ignored sender and receiver factors."

> This statement may be rephrased to say "mostly ignored" or "not focussed on", instead of "have ignored". For example, Whiting et al. (2012) and Lam et al. (2019) and have considered sender aspect (i.e., voicer credibility), and Schreurs et al. (2020) have considered relational aspects of sender and receiver (i.e., psychological distance). The authors may also benefit by referring to Ng et al. (2019).

Pages 5-6, Lines 103-107
"In prior research, most scholars believed that the influence of mood could be transferred only from high-power to low-power individuals rather than the other way around. That belief led to extensive efforts to determine the role of employee mood in upward communication [e.g., 18,19] and little attention to leader mood in downward communication."

> The research cited are examples of "leader mood in downward communication". These citations suggest that you were trying to argue the opposite. Please check and rephrase the above sentence appropriately to clearly convey your argument.

Page 21, Line 346
"Prior work on voice has begun to explore employee mood but has tended to minimize leader mood."

> Please elaborate what you mean by "tended to minimize leader mood".

Page 23, Lines 383-393

> The authors may benefit by referring to Ward et al. (2016) in relation to their discussion on impact of high-context and low-context cultures on voice.

I hope that these suggestions and concerns may help the authors revise and improve the paper.

RECOMMNEDATION: Accept with minor changes.

REFERENCES:

Lam, C. F., Lee, C., & Sui, Y. (2019). Say it as it is: Consequences of voice directness, voice politeness, and voicer credibility on voice endorsement. Journal of Applied Psychology, 104(5), 642–658. https://doi.org/10.1037/apl0000358

Ng, K. Y., Van Dyne, L., & Ang, S. (2019). Speaking out and speaking up in multicultural settings: A two-study examination of cultural intelligence and voice behavior. Organizational Behavior and Human Decision Processes, 151, 150-159.

Schreurs, B., Hamstra, M. R., & Davidson, T. (2020). What's in a word? Using construal-level theory to predict voice endorsement. European Journal of Work and Organizational Psychology, 29(1), 93-105.

Ward, A. K., Ravlin, E., Klaas, B., Ployhart, R., & Buchan, N. (2016). When Do High-Context Communicators Speak Up? Exploring Contextual Communication Orientation and Employee Voice. Journal of Applied Psychology, 101(10), 1498-1511.

Whiting, S. W., Maynes, T. D., Podsakoff, N. P., & Podsakoff, P. M. (2012). Effects of message, source, and context on evaluations of employee voice behavior. The Journal of applied psychology, 97(1), 159-182.
